# Possible Role of CYP450 Generated Omega-3/Omega-6 PUFA Metabolites in the Modulation of Blood Pressure and Vascular Function in Obese Children

**DOI:** 10.3390/nu10111689

**Published:** 2018-11-05

**Authors:** Sara Bonafini, Alice Giontella, Angela Tagetti, Denise Marcon, Martina Montagnana, Marco Benati, Rossella Gaudino, Paolo Cavarzere, Mirjam Karber, Michael Rothe, Pietro Minuz, Franco Antoniazzi, Claudio Maffeis, Wolf Hagen Schunck, Cristiano Fava

**Affiliations:** 1 Department of Medicine, University of Verona, 37129 Verona, Italy; bonafinisara@gmail.com (S.B.); alice.giontella@gmail.com (A.G.); angela.tagetti@libero.it (A.T.); denise.m@hotmail.com (D.M.); pietro.minuz@univr.it (P.M.); 2 Department of Neurosciences, Biomedicine and Movement Sciences, University of Verona, 37129 Verona, Italy; martina.montagnana@univr.it (M.M.); marco.benati@univr.it (M.B.); 3 Department of Surgery, Dentistry, Paediatrics and Gynaecology, University of Verona, 37129 Verona, Italy; rossella.gaudino@univr.it (R.G.); paolo.cavarzere@ospedaleuniverona.it (P.C.); franco.antoniazzi@univr.it (F.A.); claudio.maffeis@univr.it (C.M.); 4 Berlin Institute of Health (BIH), 10178 Berlin, Germany; mirjam.karber@charite.de; 5 Lipidomix, 13125 Berlin, Germany; michael.rothe@lipidomix.de; 6 Max Delbrueck Center for Molecular Medicine, 13092 Berlin, Germany; schunck@mdc-berlin.de

**Keywords:** CYP450 eicosanoids, omega-3 PUFA, omega-6 PUFA, blood pressure, hemodynamics, children, EETs, EEQs

## Abstract

Obesity is often accompanied by metabolic and haemodynamic disorders such as hypertension, even during childhood. Arachidonic acid (AA) is metabolized by cytochrome P450 (CYP450) enzymes to epoxyeicosatrienoic acids (EETs) and 20-hydroxyeicosatetraenoic acid (20-HETE), vasoactive and natriuretic metabolites that contribute to blood pressure (BP) regulation. Eicosapentaenoic acid (EPA) and docosahexaenoic acid (DHA) omega-3 polyunsaturated fatty acids may compete with AA for CYP450-dependent bioactive lipid mediator formation. We aimed at investigating the role of AA, EPA and DHA and their CYP450-dependent metabolites in BP control and vascular function in 66 overweight/obese children. Fatty acid profile moderately correlated with the corresponding CYP450-derived metabolites but their levels did not differ between children with normal BP (NBP) and high BP (HBP), except for higher EPA-derived epoxyeicosatetraenoic acids (EEQs) and their diols in HBP group, in which also the estimated CYP450-epoxygenase activity was higher. In the HBP group, EPA inversely correlated with BP, EEQs inversely correlated both with systolic BP and carotid Intima-Media Thickness (cIMT). The DHA-derived epoxydocosapentaenoic acids (EDPs) were inversely correlated with diastolic BP. Omega-3 derived epoxymetabolites appeared beneficially associated with BP and vascular structure/function only in obese children with HBP. Further investigations are needed to clarify the role of omega-3/omega-6 epoxymetabolites in children’s hemodynamics.

## 1. Introduction

The prevalence of overweight and obesity in children and adolescents has been increasing in the last few decades worldwide. Childhood obesity can have serious short- and long-term consequences, including metabolic and hemodynamic complications, such as hypertension, insulin resistance and dyslipidaemia [1].

Beside the impact of body mass index (BMI) per se on blood pressure (BP), several mechanisms are implicated in hemodynamic control. Dietary habits, in particular, lipid intake can influence not only body weight but also haemodynamics [2,3]. Indeed, in the last years many studies assessed the role of different polyunsaturated fatty acids (PUFA), such as arachidonic acid (AA), an omega-6 PUFA and eicosapentaenoic acid (EPA) and docosahexaenoic acid (DHA), which belong to omega-3 PUFA family, indicating a beneficial cardiovascular effect of omega-3 PUFA [4,5], whereas it is not clear whether omega-6 PUFA are cardioprotective or not [6,7]. Several trials and meta-analyses indicated a possible beneficial effect of omega-3 PUFA supplementation on BP control, although the effect is modest, and the results of the studies are not always consistent [5,8,9]. Furthermore, omega-3 PUFA may improve vascular function, that is, arterial stiffness and endothelial function, as supported by some studies and meta-analysis [10,11,12], suggesting a possible, at least partial, BP lowering effect of omega-3 PUFA. We have recently shown, in the same study population, that omega-6 PUFA and in particular AA are inversely associated with several features of the metabolic syndrome in a sample of obese children, suggesting their possible protective role [13].

AA, EPA and DHA can be metabolized via cytochrome P-450 (CYP450) into lipid mediators, which can have profound effects on blood pressure control [2,14], AA can be metabolized by different CYP450 enzymes leading to the formation of several compounds: 5,6-, 8,9-, 11,12-, 14,15-epoxyeicosatrienoic acids (EETs) via epoxygenase and for example, 20-hydroxyeicosatetraenoic acid (20-HETE) via hydroxylases. In general, EETs have been shown to play a protective role in cardiovascular diseases through their anti-inflammatory, vasodilating and sodium excreting effects [15,16]. They are further metabolized by soluble epoxide hydrolase (sEH) to dihydroxyeicosatrienoic acids (DHETs), which are less potent or even inactive compounds. Instead, 20-HETE exerts a natriuretic action at kidney level, but, in the systemic vasculature, it induces vasoconstriction [17,18]. Linoleic acid (LA) is also metabolized by CYP450 epoxygenase to 9,10- and 12,13-epoxyoctadecenoic acids (EpOMEs), which are converted by sEH to 9,10- and 12,13-dihydroxyoctadecenoic acids (DiHOMEs). They have potential toxic cardiovascular effects, probably mediated by mitochondrial dysfunction and may affect the cardiac inotropic response but the evidences are rare and diverging [19,20]. Epoxyeicosatetraenoic acids (EEQs), yielded from EPA, have vasodilating and anti-inflammatory properties and are further metabolized by sEH to diydroxxyeicosatetraenoic acids (DiHETEs) [21,22,23]. Epoxydocosapentaenoic acids (EDPs), derived from DHA via CYP-epoxygenase, are potent vasodilators [23]; they are hydrolysed by sEH to dihydroxydocosapentaenoic acids (DiHDPAs). Preliminary studies in animal models have shown that the epoxymetabolites derived from EPA and DHA have a vasodilating action that largely exceeds that of the AA-derived epoxides [24,25]. EPA and DHA can also be metabolized by CYP-hydroxylase producing for example, 20-hydroxyeicosapentaenoic acid (20-HEPE) and 22-hydroxydocosahexaenoic acid (22-HDHA) respectively, whose biological activities are still largely unknown.

Thus, the aim of the present study was to evaluate the possible association between the omega-3 and omega-6 PUFA metabolites via CYP450-epoxygenase/sEH in the modulation of haemodynamic parameters and especially BP, in a sample of overweight/obese children.

## 2. Materials and Methods

### 2.1. Patients and Study Design

Obese children were recruited consequently from October 2012 to September 2014, coming from the “Paediatric Obesity Outpatients Unit” of the University Hospital of Verona and of the “Local Health Unit n. 20” of Verona. Inclusion criteria were: children and adolescents aged 5–18 years; overweight or obesity (BMI ≥ 85th and 95th percentile for sex and age, respectively). We excluded children with hepatic or renal chronic diseases, malignancies, diabetes mellitus, lipid-lowering or antihypertensive therapy, secondary causes of obesity.

The study was conducted according to a cross-sectional observational design.

The study was approved by the Ethical Committee of the University Hospital of Verona (CE n. 2218) and written informed consent was obtained from each participant’s parents.

### 2.2. Anthropometric, Blood Pressure and Vascular Assessments

Each child was evaluated in a single occasion, between 8:00 and 9:00 a.m. A questionnaire was administered to patients and to their parents, dealing with medical history, family history, physiological and pathological information and use of drugs. Then, the participants underwent a physical examination. They were advised not to engage in strenuous exercise and to avoid consuming caffeine containing beverages within 12 h preceding the vascular studies.

During the visit, blood pressure was measured with a semiautomatic oscillometric device (TM-2551, A&D instruments Ltd., Abingdon Oxford, UK) 3 times, 3 min apart with the patient lying supine for at least 10 min before the first measurement in a room with controlled temperature (22–24 °C). The mean value of the 3 clinostatic measurements were calculated and considered for z-score and percentile calculation. Afterward, BP levels were confirmed by a measurement in the sitting position by the oscillometric device and by auscultatory method. Ambulatory blood pressure measurement (ABPM) was recorded with an oscillometric device (Spacelabs 90217; Spacelabs Inc., Issaquah, WA, USA), which measured BP every 15 min during the day and ever 30 min during the night. Children and parents recorded physical activities, resting and sleeping time and symptoms on a dedicated diary. After recording, the daytime and night-time periods (set to default at 7:00 A.M. and 10:00 P.M., respectively) were adapted to “real” awake and sleep times according to what was declared in the activity diary.

All values derived from BP measurements were transformed in z-score and percentile, according to normative values [26,27,28]. The 95th of office and ambulatory BP measurements was used as cut-off for hypertension, according to current European guidelines [27].

Body weight, height and waist and hip circumferences were measured with the patient wearing light clothes. Body weight was measured by a calibrated balance and height by a calibrated stadiometer.

Body mass index (BMI) calculated as weight in kg divided by the square of height in m; waist/hip ratio was calculated as waist circumference in cm divided by hip circumference in cm and waist/height ratio (WHtR) was calculated as waist circumference in cm divided by height in cm.

Waist circumference was transformed in z-score and percentile according to normative values [29]. Overweight or obesity were defined for BMI ≥ 90th and 95th percentile for sex and age, respectively [30]. WHO reference for BMI was used for categorizing children into the overweight and obese groups [31].

Carotid Intima-media Thickness (cIMT) was assessed by ultrasound of carotid arteries (LogiQ P5 Pro) and the cIMT was estimated tracking the artery wall in the last centimetre of the common carotid artery and calculated by a dedicated software (Multimedia Video Engine II (MVE2) DSP Lab., Pisa CNR, Italy). The relative z-score and percentile were calculated according to reference values [32].

Endothelial function was assessed by ultrasound of the brachial artery using the Flow Mediated Dilatation (FMD) technique according to international guidelines and with the aid of a dedicated hardware (Multimedia Video Engine II (MVE2) DSP Lab., Pisa CNR, Italy) [33]. Common carotid artery distensibility (DC) was calculated as: DC = ΔA/(A × ΔP) where A is the diastolic lumen area, ΔA is the stroke change in lumen area and ΔP is pulse pressure (PP). Changes in diameters were detected using ultrasound B-mode image sequences of the right and left common carotid arteries acquired at different steps and analysed by the above-mentioned automatic system [34]. The relative z-score and percentile were calculated according to reference values [32].

### 2.3. CYP-Derived Eicosanoids Measurement

Plasma samples were subjected to alkaline hydrolysis and subsequent solid phase extraction was performed as described previously [35].

In brief, 500 µL Methanol, 300 µL 10 molar sodium hydroxide and deuterated internal standards were added to 500 µL Plasma. The samples were hydrolysed for 30 min at 60 °C. The solution containing free fatty acids and metabolites were neutralized with acetic acid and adjusted to pH = 6.2.

A solid phase extraction procedure using Agilent Bond-Elut-Certify II was performed as formerly described by Rivera [36].

The LC-ESI-MS/MS method for determination of metabolites was performed by the Lipidomix GmbH, Berlin Germany, as described previously [35]. The plasma levels of individual metabolites are given in ng/mL.

Moreover, biomarkers reflecting the endogenous CYP450 epoxygenase and sEH activities were estimated by calculating the sum of epoxymetabolites plus their corresponding diols or the ratio between diols and epoxymetabolites.

### 2.4. Red Blood Cell Membrane Fatty Acids Measurement

EDTA-blood tubes were centrifuged, plasma and buffy coat taken off and erythrocytes frozen at −80 °C until analysis. Erythrocyte fatty acid composition was analysed using the HS-Omega-3 Index^®^ methodology as previously described [37,38]. Fatty acid methyl esters were generated from erythrocytes by acid transesterification and analysed by gas chromatography using a GC2010 Gas Chromatograph (Shimadzu, Duisburg, Germany) equipped with a SP2560, 100-m column (Supelco, Bellefonte, PA, USA) using hydrogen as carrier gas. Fatty acids were identified by comparison with a standard mixture of fatty acids characteristic of erythrocytes. A total of 26 fatty acids were identified and quantified.

Results are given as percentage of total identified fatty acids after response factor correction. The coefficient of variation for EPA plus DHA and for most other fatty acids was 4%. Analyses were quality-controlled according to DIN ISO 15189. All fatty acid determinations were performed by Omegametrix GmbH, Munich, Germany.

### 2.5. Statistics

Data are presented as the median and range unless otherwise stated. The statistical analysis was performed using the software Statistical Package for Social Sciences software (SPSS/PC for Windows version 21.0; IBM, Armonk, New York, NY, USA). Bivariate nonparametric correlations were estimated by Spearman coefficient (r_S_).

Differences in the measured parameters between normotensive and hypertensive children were analysed by nonparametric (Wilcoxon-Mann-Witney U) tests. A two-tailed test with a *p* < 0.05 was considered statistically significant. In order to take into account the multiple comparisons, along with original p-values, the false discovery rate (FDR) adjusted *p*-values were also calculated and reported in the tables, where appropriate.

## 3. Results

### 3.1. Patient Characteristics

From the whole study consisting of 72 children we included 66 children (females *n*: 28; 42%) with full phenotypic data about eicosanoids, red blood cell membrane FA, ABPM and vascular tests in the present study.

The collection lasted from October 2012 to October 2014 and included children are 5 to 17 years old. Four children were overweight (BMI > 90th) and 62 children were obese (BMI > 95th percentile for sex and age). All children had a central distribution of adiposity (percentile of waist circumference > 90th percentile).

On the basis of ABPM, we divided the population in two subgroups: high blood pressure (HBP) (*n*: 17; 26%) and normal blood pressure (NBP) (*n*: 49; 74%). In an exploratory analysis we investigated the correlations of fatty acids and their metabolites with BP and vascular function in each subgroup, in order to check different associations related to the hypertensive status.

General characteristics of the obese children split by gender and NBP and HBP, diagnosed on the basis of ABPM (daytime, night-time or 24-hours systolic BP(SBP) or diastolic BP (DBP) ≥ 95th percentile), are detailed in Table 1. The characteristics of the children divided according to pubertal status are listed in Table S1.

Girls showed higher BP at ABPM compared to boys. Omega-3 Index, a marker of dietary intake of long-chain omega-3 PUFA, was 4.61% (2.87–6.61%).

### 3.2. Omega-6 PUFA

#### 3.2.1. LA and CYP450-Derived Eicosanoids

##### Associations of LA with Clinical Features and with its Metabolites

**Figure 1:**
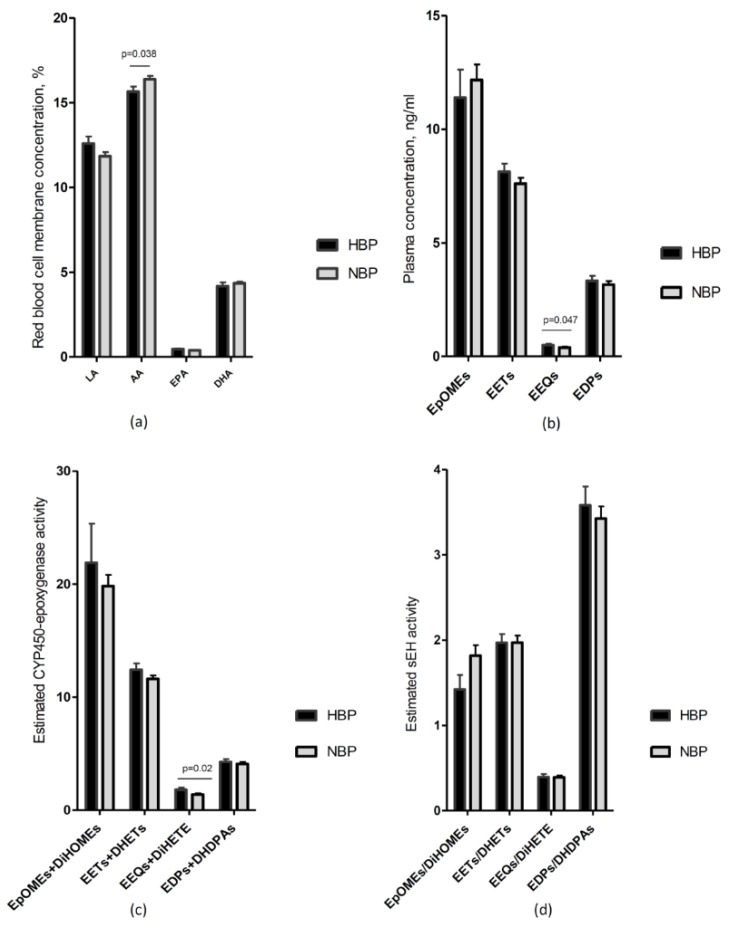
Comparison of fatty acids and their metabolites via CYP450-epoxygenase in HBP and NBP obese children. The histograms represent the comparison between HBP and NBP obese children for: (**a**) concentration of fatty acids in red blood cell membranes, that is, LA, AA, EPA and DHA; (**b**) plasma concentration of the CYP450 epoxygenase generated metabolites, that is, EpOMEs, EETs, EEQs and EDPs; (**c**) the estimated CYP450 epoxygenase activity, calculated as the sum of epoxymetabolites plus their corresponding diols; (**d**) the estimated sEH activity, calculated as the ratio between plasma concentrations (ng/mL) of diols and epoxymetabolites. Data are presented as mean and SEM. LA: Linoleic acid; AA: Arachidonic acid; EPA: Eicosapentaenoic acid; DHA: Docosahexaenoic acid; EpOME: epoxyoctadecenoic acid; DiHOME: dihydroxyoctadecenoic acid; EET: epoxyeicosatrienoic acid; DHET: dihydroxyeicosatrienoic acid; EEQ: epoxyeicosatetraenoic acid; DiHETE: dihydroxyeicosatetraenoic acid; EDP: epoxydocosapentaenoic acid; DiHDPA: dihydroxydocosapentaenoic acid; HBP: high blood pressure subgroup; NBP: normal blood pressure subgroup; sEH: soluble epoxide hydrolase; *p*: *p*-value. Vertical lines represent the standard errors.

**Figure 2:**
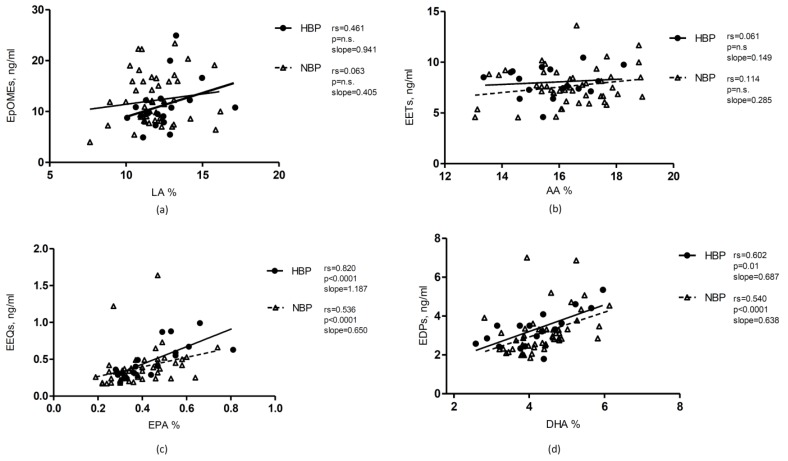
Correlations between fatty acids and their metabolite via CYP450-epoxygenase. The graphs represent the correlations of the fatty acids with their corresponding epoxymetabolites. The two subgroups of obese children with high (HBP) and normal blood pressure (NBP) are differently graphically depicted. The panels represent: (**a**) the correlation of LA with EpOMEs, which was stronger in HBP children; (**b**) the association of AA with EETs; (**c**) the correlation of EPA with EEQs, which was stronger in HBP children; (**d**) the association of DHA with EDPs. LA: Linoleic acid; AA: Arachidonic acid; EPA: Eicosapentaenoic acid; DHA: Docosahexaenoic acid; EpOME: epoxyoctadecenoic acid; EET: epoxyeicosatrienoic acid; EEQ: epoxyeicosatetraenoic acid; EDP:epoxydocosapentaenoic acid; HBP: high blood pressure subgroup; NBP: normal blood pressure subgroup, *p*: *p*-value; r_S_: Spearman correlation coefficient; n.s.: not significant.

LA was similarly distributed according to gender and hypertensive status (Table 1 and Figure 1). LA directly correlated with DiHOMEs in the whole population and in particular in HBP children (Table 2 and Figure 2). LA also directly correlates with the estimated CYP450 epoxygenase activity in HBP (Figure S1).

##### CYP450-Derived Eicosanoids of LA and Their Associations with Clinical Features

LA did not show any significant correlation with BP (Table 3), whereas DiHOMEs, especially 9,10-DiHOME, directly correlated with DBP in the whole sample and in the NBP subgroup (Table 4). (The associations of the eicosanoids with BP percentiles are reported in Table S2). Vascular tests did not show any significant correlation with LA and its metabolites (Table 5).

#### 3.2.2. AA and CYP450-Derived Eicosanoids

##### Associations of AA with Clinical Features and with Its Metabolites

AA was higher in NBP as compared to HBP children (Table 1 and Figure 1). In the whole sample, AA was inversely correlated to ABPM and this inverse association was confirmed in the NBP subgroup (Table 3). No significant correlations were found between AA and vascular features (Table 5).

Considering the metabolic pathway of AA via CYP450 and sEH, AA was not significantly correlated with its metabolites via CYP450/sEH neither in the whole population nor in the subgroups of HBP and NBP children (Table 2 and Figure 2). AA did not show any significant difference also with the estimated CYP450 epoxygenase activity (Figure S1).

##### CYP450-Derived Eicosanoids of AA and Their Associations with Clinical Features

EETs and DHETs, the epoxymetabolites and the corresponding diols derived from AA respectively, did not show any significant association with BP and vascular function markers (Table 4 and Table 5. The associations of the eicosanoids with BP percentiles are detailed in Table S2). Twenty-hydroxyeicosatetraenoiic acid (20-HETE), the hydroxymetabolite of AA, did not differ between HBP and NBP subgroups (Table 1) and did not show any significant correlation with BP and vascular features.

### 3.3. Omega-3 PUFA

#### 3.3.1. EPA and CYP450-Derived Eicosanoids

##### Associations of EPA with Clinical Features and with Its Metabolites

The amount of EPA in red blood cell membrane was similar in both groups of HBP and NBP obese children (Table 1 and Figure 1). EPA inversely correlated with office BP and with cIMT in the HBP subgroup and the inverse correlation with cIMT was significant also in the whole population (Table 3 and Table 5).

EPA was directly correlated with its epoxymetabolites and the corresponding diols, especially the 14,15-isomer, in the whole sample and in both subgroups (Table 2). In the subgroup of HBP children the strength and the slope of the correlations between the precursor and the products were higher and EEQs and DiHETEs were more elevated than in NBP (Figure 2). Moreover, the estimated CYP450 activity (sum of EEQs and DiHETEs) was higher in the HBP than in NBP children (Figure 1 panel (c)) and EPA correlated stronger with the estimated CYP450 activity in the HBP than in NBP subgroup, whereas the estimated sEH activity did not differ between the two groups (Table 2 and Figure S1).

##### CYP450-Derived Eicosanoids of EPA and Their Associations with Clinical Features

**Figure 3:**
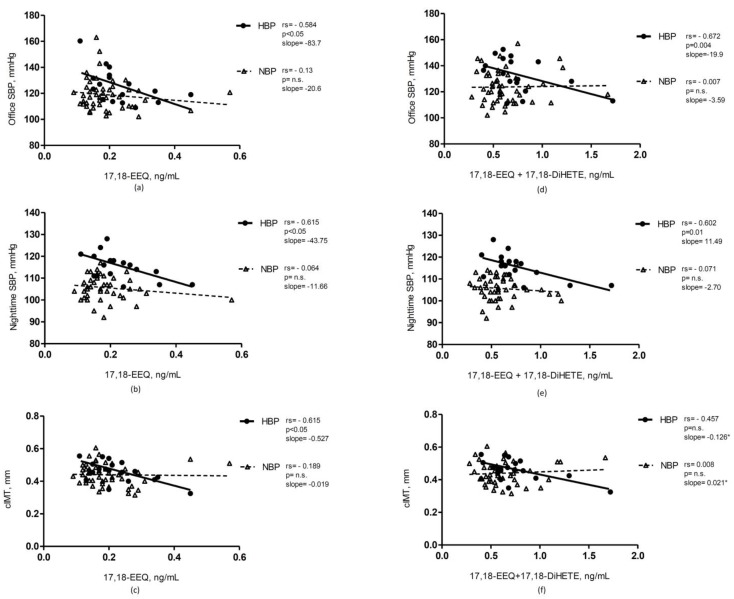
Correlations of EEQ with BP and cIMT in the high blood pressure (HBP) and normal blood pressure (NBP) obese children subgroups. The first column represents the correlation of 17,18-EEQ with: (**a**) office SBP; (**b**) night-time SBP; (**c**) cIMT. The second column represents the correlation of the estimated CYP450 epoxygenase activity, as calculated as the sum of epoxymetabolites plus the corresponding diols, with: (**d**) office SBP; (**e**) night-time SBP; (**f**) cIMT. * there is a significantly difference between the slopes of the two subgroups, *p* < 0.05. The correlations in all panels are stronger in HBP than in NBP children. The two subgroups of obese children with high and normal blood pressure are differently graphically depicted. cIMT: carotid intima-media thickness; EEQ: epoxyeicosatetraenoic acid; DiHETE: dihydroxyeicosatetraenoic acid; HBP: high blood pressure subgroup; NBP: normal blood pressure subgroup; SBP: systolic blood pressure, *p*: *p*-value; r_S_: Spearman correlation coefficient.

EEQs and DiHETEs inversely correlated with office and ambulatory BP, in particular 11,12-EEQ and 17,18-DiHETE (Table 4 and Figure 3. The associations of the eicosanoids with BP percentiles are reported in Table S2). In the whole sample, only 8,9-EEQ showed an inverse correlation with office DBP (Table 4.). The epoxymetabolites of EPA inversely correlated also with cIMT, in particular 14,15- and 17,18- EEQ (Figure 3) and these associations were more evident in the HBP group, in which also 5,6-DiHETE sowed an inverse correlation with cIMT. In this subgroup 11,12-EEQ was also directly correlated with carotid distensibility (Table 5). Plasma concentration of twenty-hydroxyeicosapentaenoic acid (20-HEPE), the hydroxymetabolite of EPA, was under the limit of detection.

#### 3.3.2. DHA and CYP450-Derived Eicosanoids

##### Associations of DHA with Clinical Features and with Its Metabolites

DHA had a similar distribution among genders and hypertensive status (Table 1) and was associated neither with BP nor with the main vascular features (Table 3 and Table 5).

DHA was directly correlated with its epoxymetabolites, the EDPs, in the whole population and in both subgroups and was directly correlated with DiHDPAs in particular in NBP children (Table 2 and Figure 2). DHA directly correlated with the estimated CYP450 epoxygenase activity in both subgroups (Figure S1).

##### CYP450-Derived Eicosanoids of EPA and Their Associations with Clinical Features

EDPs and DiHDPAs were not associated with BP and vascular features, except an inverse correlation of most isomers of EDPs with office SBP in HBP children (Table 4. The associations of the eicosanoids with BP percentiles are detailed in Table S2). Plasma concentrations of 22-hydroxydocosahexaenoic acid (22-HDHA) did not differ between HBP and NBP subgroups (Table 1) and did not show any significant correlation with BP and vascular characteristics, except an inverse correlation with cIMT (r_S_ = −0.292, *p* < 0.01) and the relative z-score (r_S_ = −0.297, *p* < 0.01) in the NBP subgroup.

Most of the above-mentioned significant correlations remained significant after adjustment for sex, age and pubertal status, as indicated in the relative tables. After *False Discovery Rate* (FDR) correction only most of the correlations between the precursors and the respective products remained significant.

## 4. Discussion

Our main hypothesis was that several PUFA, mainly assumed by the diet, could affect hemodynamics and in particular blood pressure, through the formation of specific PUFA-derived lipid mediators generated via the CYP450-epoxygenase/sEH pathway. Scarce is the evidence linking the amount of fatty acids introduced by diet and their metabolites via CYP450/sEH with hemodynamics in humans and, to our knowledge, this is the first study that investigated this link in children. Indeed, red blood cell membrane fatty acids are a reliable marker of dietary intake, especially for essential fatty acids, reflecting the preceding intake of the fatty acid [38].

A complex link between PUFA and BP is supported by the literature, where the putative beneficial effect of omega-3 PUFA on BP and subsequent cardiovascular events is often blurred, being evident in some trials but not in others or in meta-analyses [5,39], with some studies also available in children [40,41,42]. The underlying mechanisms are multifactorial and may include an improvement in endothelial function and in arterial stiffness [10,11,12], although the relative effects of omega-3 PUFA and their CYP450 generated metabolites remain not completely understood.

Despite plenty of studies in animal models [43], especially rats, the evidence that EETs could affect BP in humans is scanty. In particular, our group found lower plasma EETs in patients affected by renovascular hypertension as compared to essential hypertension and controls [44] and an augmented production of EETs in plasma and placentas obtained by preeclamptic women [45,46]. Surprisingly, we did not find any association of EETs neither with BP nor vascular function in this sample of obese children.

Little is known about the specific actions of the EPA/DHA-derived metabolites via CYP450/sEH on hemodynamic modulation both in animal models and humans but a few studies support a protective effect on blood pressure, at least for some single isomers [23,25] suggesting that their actions could be even more potent with respect to EETs.

In this study, we found interesting correlations between EPA and DHA and the corresponding metabolites via CYP450-epoxygenase, supporting the hypothesis that a dietary assumption of specific omega-3 PUFA (or at least a higher storage in cellular membranes) drives a higher production of their metabolites. Interestingly, these metabolic steps appear different in HBP and in NBP children. Indeed, the association between precursor and products, especially for EPA metabolism, is stronger and steeper in HBP than in NBP and, moreover, also the estimated CYP450-epoxygenase activity (sum of epoxides and diols) itself is higher in HBP children whereas no difference in the estimated sEH activity (epoxide/diol ratio) was evident between the two subgroups. These data suggest that in HBP obese children the CYP450-epoxygenase, in particular for EPA, might have a different efficacy or regulation as compared to NBP. We could hypothesize that in obese children with HBP is a modulation in CYP450-epoxygenase activity, rather than in sEH activity, leading to an enhanced formation of the epoxymetabolites, which are supposed to have more favourable cardiovascular effects.

Furthermore, the exploratory analyses in the subgroup of HBP children, seems to reveal a potential role of some metabolites (especially EEQs and EDPs) on BP. Especially EEQs and in particular 14,15- and 17,18- EEQ, showed inverse associations with several BP measurements and with the markers of vascular structure and function (namely, cIMT and distensibility) in HBP obese children. Even if this is only an exploratory analysis and should be examined with caution, it is compatible with the hypothesis that the effect of endothelium derived hyperpolarizing factors (such as EETs/EEQs/EDPs) is detectable only in circumstances when the effect of nitric oxide (NO) is altered [47,48]. Indeed, some of the beneficial cardiovascular effects of long-chain omega-3 PUFA and especially EPA, at least those mediated by CYP450/sEH metabolites, could act as compensatory mechanism and thus being more evident in more disadvantageous conditions, that is, obesity together with high blood pressure. Only a few studies addressed this issue: a study in transgenic mice with endothelial expression of the human CYP2J2 and CYP2C8 demonstrated that increased endothelial CYP450 epoxygenase expression reduced BP increase especially when nitric oxide synthase and cyclooxygenase were inhibited [49]. Data from studies in humans also support the hypothesis that the CYP450 pathways compensate the impairment of vasodilation due to classical pathways, like NO, for example in hypertensive patients [47] and in subjects with hyperpathyroidism, which is generally associated with high blood pressure and an increased CV risk [50].

In line with our observations, also some other studies suggest that EPA and DHA exert a stronger effect in hypertensive as compared to normotensive subjects, as suggested also by meta-analyses [5,39,51]. Interestingly, the inverse association of 14,15- and 17,18-EEQ with cIMT, is even stronger in the HBP subgroup compared to the whole group as well.

The observational study design and the exploratory analyses suggest looking at all these associations with caution. Anyhow, we hypothesize that EPA could exert a somehow protective action on blood pressure through their metabolites via CYP450-epoxygenase/sEH, probably mediated by a beneficial influence on vascular structure and function and this effect could be stronger in HBP obese children than in NBP. The stronger correlations of EPA with its metabolites in HBP rather than in NBP children support the hypothesis that their effect becomes more important when BP is higher.

Interestingly, DiHOME, the diols derived from LA via CYP450 and sEH metabolism, showed a direct correlation with diastolic BP in NBP but not in HBP, suggesting that they negatively affect vascular stiffness and BP but only in normotensive obese children. Very little is known about the actions of EpOMEs and DiHOMEs; first data indicated a toxic effect of EpOME and probably of DiHOME that could be dose-dependent; they also could affect cardiac contractility but the results are not always consistent [12,13]. On the other hand, a recent study has proposed a possible protective effect of 12,13-DiHOME on metabolic profile, due to its action on brown adipose tissue uptake of fatty acids [52]. Furthermore, an epoxy-keto derivative of LA has been identified as a possible stimulating factor for aldosterone secretion but its precursor and its metabolic pathway is not known [53]. Furthermore, we found that LA was inversely correlated to BP in HBP subjects, suggesting a possible beneficial effect of LA on BP. These data are not easy to interpret, considering the results of the metabolite of LA, especially the possible positive role of 12,13-DiHOME. Anyhow, it should be considered that from each fatty acid derives a range of lipid mediators, which can have different actions. Moreover, also in a previous animal model a discrepancy has been found regarding the effect of dietary intake of fatty acids, that is, LA and ALA, on the composition of PUFA and their related metabolites [54].

As mentioned above, the present study has limitations: the lack of data about the total food intake of fatty acids and the amount of fatty acids in the diet, the sample size is relatively low, which can primarily expose a problem of statistical power; after statistical adjustment for multiple testing only several correlations remained significant. Thus, considering also the observational design of the study, the results should be regarded as exploratory and need confirmation in new studies. Furthermore, we decided to define hypertension on the basis of a single ABPM registration, considering it being more accurate to establish hypertension based on a single exam. Finally, these results need to be confirmed in other studies analysing also samples of children of different ages and body size, including non-obese children and different ethnic groups.

Anyhow, we obtained also several office BP measurements, both in the supine position and in the recommended sitting position, which strengthened the results derived from ABPM.

Globally, our data suggest that single lipid mediators may exert specific actions in hemodynamic control in obese children, that may be different in hypertensive rather than in normotensive children. What remains to be understood are the regulatory mechanisms that can modulate the metabolic pathways of the fatty acids, leading to the production of specific lipid mediators. Moreover, also the relations, or the competition, between different metabolic pathways might affect the productions of active metabolites, thus influencing the final effect [2]. Furthermore, the omega-3 index of the obese children included in this study was quite low (mean: 4.6%), far below the proposed target of 8% or more, which is suggested as CV protective level by the evaluation of epidemiological and clinical studies [55,56]. Thus, another question that should be addressed by future studies is whether obese children with HBP may benefit from dietary EPA/DHA supplementation.

In conclusion, this study sets out the steps to further investigate, in children as well in adults, the metabolism of dietary fatty acids, especially via CYP450 and their possible influence on hemodynamics and BP control.

## Figures and Tables

**Table 1:** General characteristics of the population divided according to gender and blood pressure status.

	Male (*n* = 38)	Female (*n* = 28)		NBP (*n* = 49)	HBP (*n* = 17)	
Variable	Median (Range)	Median (Range)	*p*-Value *	Median (Range)	Median (Range)	*p*-Value *
Age, years	11.5 (10.0–14.0)	11.0 (9.0–13.0)	0.187	12.0 (10.0–13.5)	11.0 (8.5–12.5)	0.181
BMI, Kg/m^2^	28.3 (25.4–31.0)	29.3 (26.1–33.2)	0.392	28.4 (25.7–31.0)	30.4 (26.7–35.3)	0.125
BMI percentile	98.3 (97.2–99.0)	98.7 (97.8–99.3)	0.238	98.0 (97.3–99.0)	99.1 (98.3–99.5)	0.011
Office-SBP, mmHg	119.0 (112.9–125)	116.3 (109.8–129)	0.471	117.0 (110.2–110.2)	123.3 (114.5–133.2)	0.031
Office-SBP percentile	83.1 (64.9–94.8)	79.9 (67.7–96.4)	0.697	77.7 (64.7–93.7)	95.6 (76.7–99.3)	0.012
Office-DBP, mmHg	68.3 (63.3–76.1)	66.7 (64.1–72.7)	0.673	66.7 (63.7–72)	75.0 (63.7–77.3)	0.179
Office-DBP percentile	65.1 (43.9–80.7)	66.1 (50.8–77.9)	0.577	64.3 (46.9–76.2)	83.0 (51.4–87.2)	0.033
24 h-SBP, mmHg	118.0 (114–121)	112.0 (107–116)	0.001 °	114.0 (109–118)	121.0 (118.5–128.0)	<0.001 °
24 h-SBP, percentile	73.7 (50.6–82.6)	54.4 (35.3–79.8)	0.169	51.7 (37.8–74.6)	90.4 (80.0–98.8)	<0.001 °
24 h-DBP, mmHg	68.0 (64.0–70.3)	64.0 (61.3–67.0)	0.001 °	65.0 (62.0–68.5)	70.0 (65.5–74.5)	0.002 °
24 h-DBP, percentile	57.4 (28.5–74.5)	34.5 (18.7–50.6)	0.007	35.1 (20.0–57.4)	69.9 (42.5–94.5)	0.001 °
cIMT, mm	0.46 (0.42–0.51)	0.41 (0.39–0.47)	0.032	0.44 (0.40–0.48)	0.46 (0.42–0.51)	0.179
cIMT, percentile	98.4 (80.3–99.7)	82.3 (60.4–96.7)	0.021	88.2 (59.6–99.1)	96.5 (82.2–99.7)	0.207
cDC, 10^−3^/Kpa	39.7 (33.5–46.4)	43.8 (34.9–48.3)	0.199	42.0 (34.7–48.5)	37.3 (33.0–42.5)	0.127
cDC, percentile	6.4 (2.4–23.2)	12.8 (2.2–27.0)	0.249	12.3 (2.9–27.0)	4.2 (2.2–13.2)	0.110
Glucose, mg/dL	88.0 (84.0–93.0)	85.0 (79.0–88.0)	0.026	86.0 (83–90)	87.0 (82–93.8)	0.855
Insulin, uU/mL	18.5 (12.4–27.5)	17.8 (13.0–28.2)	0.887	17.7 (11.8–27.6)	24.1 (17.1–30.0)	0.110
Cholesterol, mg/dL	160 (139.9–186.8)	165.0 (134–192)	0.536	165.0 (139–192)	151.5 (132–173.3)	0.297
Triglycerides, mg/dL	79 (55–103.5)	79 (54–100)	0.744	76.5 (54–99.8)	87 (59.5–118)	0.231
FMD, %	7.7 (5.3–10.2)	6.2 (3.4–9.6)	0.403	7.4 (4.2–10.2)	7.6 (3.2–9.8)	0.682
LA, %	11.8 (11.1–12.9)	11.8 (11.1–12.8)	0.841	11.7 (11.0–12.6)	12.4 (11.4–13.1)	0.104
AA, %	16.0 (14.9–17.1)	16.4 (15.8–17.3)	0.082	16.4 (15.6–17.5)	15.5 (14.6–16.6)	0.038
EPA, %	0.4 (0.3–0.5)	0.4 (0.3–0.5)	0.340	0.37 (0.30–0.47)	0.4 (0.3–0.6)	0.127
DHA, %	4.3 (3.8–4.7)	4.4 (3.8–5.0)	0.425	4.4 (3.8–4.8)	4.2 (3.5–4.8)	0.367
Omega-3 Index, %	4.6 (4.1–5.2)	4.6 (4.3–5.5)	0.417	4.6 (4.2–5.3)	4.5 (3.9–5.3)	0.639
EpOMEs, ng/mL	11.2 (8.2–15.9)	10.8 (8.7–15.7)	0.907	11.5 (8.4–15.9)	10.8 (8.3–12.4)	0.613
DiHOMEs, ng/mL	7.2 (5.3–9.7)	6.3 (5.3–8.8)	0.340	6.5 (5.3–9.2)	7.5 (5.6–9.6)	0.363
EpOMEs/DiHOMEs	1.6 (0.9–2.1)	1.6 (1.3–2.4)	0.347	1.7 (1.3–2.3)	1.2 (0.8–1.9)	0.093
EpOMEs+DiHOME	18.6 (14.0–25.7)	18.4 (15.3–22.2)	0.795	19.4 (14.8–23.7)	17.0 (13.4–24.2)	0.634
EETs, ng/mL	7.4 (6.6–8.9)	7.7 (6.8–8.9)	0.460	7.5 (6.6–8.5)	8.4 (7.2–9.2)	0.131
DHETs, ng/mL	4.1 (3.3–4.7)	4.0 (3.5–4.7)	0.726	4.0 (3.4–4.5)	4.0 (3.5–5.1)	0.367
EETs/DHETs	1.9 (1.6–2.4)	2.0 (1.6–1.2)	0.902	1.9 (1.6–2.3)	2.0 (1.6–2.2)	0.814
EETs+DHETs	11.7 (10.1–13.3)	12.1 (10.7–13.2)	0.448	11.8 (10.2–13.0)	12.7 (10.8–14.3)	0.110
EEQs, ng/mL	0.3 (0.2–0.4)	0.4 (0.3–0.5)	0.302	0.3 (0.2–0.5)	0.4 (0.3–0.7)	0.047
DiHETEs, ng/mL	0.9 (0.7–1.3)	0.9 (0.7–1.3)	0.568	0.9 (0.7–1.1)	1.2 (0.9–1.6)	0.021
EEQs/DiHETEs	0.33 (0.31–0.38)	0.42 (0.33–0.54)	0.004 °	0.37 (0.31–0.46)	0.34 (0.31–0.51)	0.872
EEQs+DiHETEs	1.3 (1.0–1.8)	1.3 (0.9–1.8)	0.871	1.2 (1.0–1.5)	1.8 (1.2–2.2)	0.025
EDPs, ng/mL	2.9 (2.4–3.3)	3.2 (2.7–4.2)	0.050	2.9 (2.4–3.3)	3.3 (2.5–3.9)	0.221
DiHDPAs, ng/mL	0.9 (0.8–1.0)	0.9 (0.8–1.1)	0.399	0.9 (0.8–1.1)	0.9 (0.8–1.1)	0.665
EDPs/DiHDPAs	3.3 (2.9–3.6)	3.6 (2.8–4.3)	0.186	3.4 (2.8–3.8)	3.2 (3.0–4.2)	0.878
EDPs+DiHDPAs	3.8 (3.1–4.3)	4.3 (3.5–5.3)	0.044	3.8 (3.3–4.4)	4.3 (3.3–4.9)	0.238
20-HETE	0.74 (0.61–0.86)	0.64 (0.57–0.87)	0.459	0.7 (0.58–0.84)	0.83 (0.53–0.91)	0.553
22-HDHA	0.65 (0.44–0.92)	0.66 (0.41–0.83)	0.721	0.67 (0.42–0.89)	0.64 (0.43–0.91)	0.837

BMI: body mass index, SBP: systolic blood pressure, DBP: diastolic blood pressure, cIMT: carotid intima-media thickness, cDC: carotid distensibility coefficient, FMD: flow-mediated dilation, LA: Linoleic acid, AA: Arachidonic acid, EPA: Eicosapentaenoic acid, DHA: Docosahexaenoic acid, Omega-3 Index: (EPA + DHA)/total FA × 100, EpOME: epoxyoctadecenoic acid, DiHOME: dihydroxyoctadecenoic acid, EET: epoxyeicosatrienoic acid, DHET: dihydroxyeicosatrienoic acid, EEQ: epoxyeicosatetraenoic acid, DiHETE: dihydroxyeicosatetraenoic acid, EDP: epoxydocosapentaenoic acid, DiHDPA: dihydroxydocosapentaenoic acid, 20-HETE: 20-hydroxyeicosatetraenoic acid, 22-HDHA: 22-hydroxydocosahexaenoic acid, HBP: high blood pressure subgroup, NBP: normal blood pressure subgroup, *n*: number. 20-hydroxyeicosapentaenoic acid (20-HEPE) plasma concentration was under the limit of detection. *: Mann-Whitney *U*-Test; °: *p* < 0.05 after False Discovery Rate correction.

**Table 2:** Correlations between fatty acids (precursors) and the derived eicosanoids via CYP450-epoxygenase/sEH.

	Whole Population	NBP	HBP
**LA**			
9,10-EpOME	0.102	0.064	0.434
12,13-EpOME	0.094	0.063	0.465
9,10-DiHOME	0.438 ^	0.311 *	0.674 ^
12,13-DiHOME	0.336 ^	0.265	0.659 ^
EpOME/DiHOME	−0.292 *	−0.172	−0.424
EpOME+DiHOME	0.292 *	0.234	0.718 ^
**AA**			
5,6-EET	0.029	0.152	−0.049
8,9-EET	0.061	0.162	−0.064
11,12-EET	0.049	0.096	0.177
14,15-EET	0.040	0.114	0.074
5,6-DHET	0.032	0.196	−0.251
8,9-DHET	0.105	0.201	−0.051
11,12-DHET	0.014	0.166	−0.418
14,15-DHET	0.210	0.218	0.220
EET/DHET	−0.007	−0.053	0.292
EET+DHET	0.051	0.186	−0.081
**EPA**			
8,9-EEQ	0.434 ^	0.267 °	0.784 ^^,^°
11,12-EEQ	0.575 ^	0.482 ^^,^°	0.794 ^^,^°
14,15-EEQ	0.641 ^	0.590 ^^,^°	0.808 ^^,^°
17,18-EEQ	0.552 ^	0.509 ^^,^°	0.643 ^^,^°
5,6-DiHETE	0.512 ^	0.441 ^^,^°	0.652 ^^,^°
8,9-DiHETE	0.398 ^	0.337 *^,^°	0.446
11,12-DiHETE	0.217	0.244	−0.023
14,15-DiHETE	0.464 ^	0.393 ^^,^°	0.532 *^,^°
17,18-DiHETE	0.484 ^	0.431 ^	0.440
EEQ/DiHETE	0.228	0.138	0.521 *^,^°
EEQ+DiHETE	0.605 ^	0.578 ^^,^°	0.652 ^
**DHA**			
7,8-EDP	0.482 ^ ^,^ °	0.553 ^ ^,^ °	0.666 ^°
10,11-EDP	0.375 ^ ^,^ °	0.360 *^,^°	0.569 *
13,14-EDP	0.467 ^ ^,^ °	0.515 ^ ^,^ °	0.563 *
16,17-EDP	0.502 ^ ^,^ °	0.591 ^^,^°	0.546 *
19,20-EDP	0.456 ^ ^,^ °	0.581 ^ ^,^ °	0.473
7,8-DiHDPA	0.453 ^ ^,^ °	0.521 ^ ^,^ °	0.324
10,11-DiHDPA	0.220	0.310 *^,^°	0.205
13,14-DiHDPA	0.256 *^,^°	0.325 * ^,^ °	0.290
16,17-DiHDPA	0.386 ^^,^°	0.531 ^ ^,^ °	0.086
19,20-DiHDPA	0.383 ^^,^°	0.565 ^ ^,^ °	0.010
EDP/DiHDPA	0.150	0.072	0.433
EDP+DiHDPA	0.526 *^,^°	0.617 ^ ^,^ °	0.575 *

The table shows the coefficients of correlations (r_S_) between the fatty acid precursors, namely LA, AA, EPA and DHA and their metabolites via CYP450-epoxygenase/sEH in the whole sample and in the subgroups of HBP and NBP children. The underlined correlations remained significant after adjustment for sex, age, pubertal status and BMI. The sum of epoxymetabolites and corresponding diols estimates the CYP450-epoxygenase activity, whereas their ratio estimates the sEH activity. *: *p* < 0.05; ^: *p* < 0.01; °: *p* < 0.05 after False Discovery Rate correction. NBP: normal blood pressure; HBP high blood pressure; LA: Linoleic acid; AA: Arachidonic acid; EPA: Eicosapentaenoic acid; DHA: Docosahexaenoic acid; EpHOME: epoxyoctadecenoic acid; DiHOME: dihydroxyoctadecenoic acid; EET: epoxyeicosatrienoic acid; DHET: dihydroxyeicosatrienoic acid; EEQ: epoxyeicosatetraenoic acid; DiHETE: dihydroxyeicosatetraenoic acid; EDP: epoxydocosapentaenoic acid; DiHDPA: dihydroxydocosapentaenoic acid.

**Table 3:** Correlations between red blood cell membrane fatty acids and BP in the whole study population and in the subgroups of HBP and NBP obese children.

	LA	AA	EPA	DHA
**Whole population**				
Office-SBP, mmHg	−0.091	−0.070	0.035	0.090
Office-SBP percentile	0.002	0.002	−0.049	−0.094
Office-DBP, mmHg	0.168	−0.130	−0.167	−0.106
Office-DBP percentile	0.217	−0.095	−0.175	−0.210
24 h-SBP, mmHg	0.096	−0.329 ^	0.154	0.016
24 h-SBP percentile	0.229	−0.134	0.014	−0.214
24 h-DBP, mmHg	0.171	−0.222	−0.069	−0.140
24 h-DBP percentile	0.200	−0.176	−0.092	−0.175
Day-time SBP, mmHg	0.070	−0.277 *	0.172	0.043
Day-time SBP, percentile	0.192	−0.129	0.086	−0.132
Day-time DBP, mmHg	0.136	−0.132	−0.075	−0.142
Day-time DBP, percentile	0.136	−0.092	−0.084	−0.142
Night-time SBP, mmHg	0.066	−0.327 ^	0.154	−0.001
Night-time SBP, percentile	0.213	−0.178	0.051	−0.121
Night-time DBP, mmHg	0.145	−0.359 ^	0.014	−0.046
Night-time DBP, percentile	0.159	−0.353 ^	0.043	−0.040
**HBP**				
Office-SBP, mmHg	0.013	0.088	−0.639 ^	−0.050
Office-SBP percentile	0.017	0.172	−0.609 ^	−0.113
Office-DBP, mmHg	0.206	−0.136	−0.564 *	−0.377
Office-DBP percentile	0.218	0.083	−0.668^	−0.157
24 h-SBP, mmHg	−0.425	0.220	−0.111	0.236
24 h-SBP percentile	−0.279	0.387	−0.043	0.250
24 h-DBP, mmHg	−0.278	−0.205	−0.251	0.010
24 h-DBP percentile	−0.228	−0.184	−0.269	−0.012
Day-time SBP, mmHg	−0.433	0.055	0.013	0.257
Day-time SBP, percentile	−0.277	0.306	0.037	0.257
Day-time DBP, mmHg	−0.263	−0.167	−0.164	0.001
Day-time DBP, percentile	−0.252	−0.142	−0.188	0.015
Night-time SBP, mmHg	−0.413	0.319	−0.225	0.172
Night-time SBP, percentile	−0.193	0.466	−0.029	0.076
Night-time DBP, mmHg	−0.258	−0.182	−0.427	−0.021
Night-time DBP, percentile	−0.228	−0.191	−0.426	−0.049
**NBP**				
Office-SBP, mmHg	−0.180	−0.034	0.169	0.188
Office-SBP percentile	−0.087	0.042	0.063	−0.050
Office-DBP, mmHg	0.089	−0.144	−0.049	0.025
Office-DBP percentile	0.128	−0.111	−0.079	−0.125
24 h-SBP, mmHg	0.059	−0.325 *	0.060	0.057
24 h-SBP percentile	0.217	−0.037	−0.163	−0.311 *
24 h-DBP, mmHg	0.233	−0.221	−0.131	−0.151
24 h-DBP percentile	0.268	−0.172	−0.159	−0.196
Day-time SBP, mmHg	0.062	−0.268	0.092	0.067
Day-time SBP, percentile	0.184	−0.069	−0.058	−0.160
Day-time DBP, mmHg	0.208	−0.124	−0.136	−0.176
Day-time DBP, percentile	0.209	−0.060	−0.147	−0.176
Night-time SBP, mmHg	0.052	−0.335 *	0.090	0.025
Night-time SBP, percentile	0.249	−0.154	−0.042	−0.212
Night-time DBP, mmHg	0.229	−0.340 *	−0.044	−0.025
Night-time DBP, percentile	0.251	−0.333 *	−0.027	−0.035

The table shows the spearman coefficients of correlations (r_s_) between fatty acids (precursors), namely LA, AA, EPA, DHA and office and ambulatory BP in the whole sample and in the subgroups of HBP and NBP children. The underlined correlations remained significant after adjustment for sex, age, pubertal status and BMI. *: *p* < 0.05; ^: *p* < 0.01 after False Discovery Rate correction. NBP: normal blood pressure; HBP high blood pressure; AA: arachidonic acid; DHA: docosahexaenoic acid; DBP: diastolic blood pressure; EPA: eicosapentaenoic acid; LA: linolenic acid; SBP: systolic blood pressure.

**Table 4:** Correlations of CYP450-epoxygenase/sEH metabolites of LA, AA, EPA and DHA with BP in the whole study population and in the two subgroups of high blood pressure and normal blood pressure obese children.

	Office SBP, mmHg	Office DBP, mmHg	24-h SBP, mmHg	24-h DBP, mmHg	Day-time SBP, mmHg	Day-time DBP, mmHg	Night-time SBP, mmHg	Night-time DBP, mmHg
Whole population								
EpOMEtot	−0.092	0.070	−0.025	0.069	−0.045	0.073	−0.086	−0.019
DiHOME tot	−0.060	0.279 * 9,10-DiHOME 0.319 ^	0.031	0.149	−0.053	0.052	0.103	0.252 * 9,10-DiHOME 0.256 *
EET tot	0.060	−0.008	0.019	0.037	0.004	0.040	0.017	−0.044
DHET tot	0.142	0.052	0.036	0.006	0.077	0.050	0.012	−0.020
EEQ tot	−0.120	−0.211 8,9-EEQ −0.307 *	0.045	0.030	0.038	0.021	0.052	−0.076
DiHETE tot	−0.069	−0.171	0.117	0.109	0.131	0.058	0.080	0.112
EDP tot	−0.128	−0.180	−0.013	−0.077	0.001	−0.067	−0.076	−0.140
DiHDPA tot	−0.035	−0.077	−0.028	−0.086	0.030	−0.054	−0.161	−0.134
HBP								
EpOME tot	−0.256	−0.094	−0.315	−0.291	−0.428	−0.380	−0.271	0.068
DiHOME tot	−0.104	0.232	−0.252	−0.098	−0.363	−0.123	−0.266	0.037
EET tot	−0.108	−0.287	−0.211	0.071	−0.087	0.183	−0.311	0.106
DHET tot	−0.016	−0.109	−0.034	−0.050	0.112	0.046	−0.102	−0.047
EEQ tot	−0.539 * 11,12-EEQ −0.694 ^ 17,18-EEQ −0.584 *	−0.299	−0.276 11,12-EEQ −0.492 *	−0.117	−0.080	−0.038	−0.439 11,12-EEQ −0.559 * 17,18-EEQ −0.615 ^	−0.216
DiHETE tot	−0.437 17,18-DiHETE −0.578 *	−0.120	−0.233 17,18-DiHETE −0.512 *	0.142	−0.076	0.205	−0.348 17,18-DiHETE −0.492 *	0.089
EDP tot	−0.229 7,8-EDP −0.574 * 10,11-EDP −0.506 * 16-17-EDP−0.539 *	−0.526*	−0.120	−0.284	−0.010	−0.205	−0.361	−0.240
DiHDPA tot	−0.090	−0.382	0.093	−0.183	0.277	−0.070	−0.195	−0.350
NBP								
EpOME tot	−0.048	0.148	0.065	0.147	0.070	0.206	−0.049	−0.033
DiHOME tot	−0.094	0.281 9,10-DiHOME 0.319 *	0.027	0.158	−0.054	0.062	0.141	0.313 * 9,10-DiHOME 0.312 *
EET tot	0.057	−0.003	−0.029	−0.076	−0.043	−0.064	−0.121	−0.264 8,9-EET −0.332 *
DHET tot	0.189	0.104	−0.013	0.013	0.025	0.034	−0.071	−0.044
EEQ tot	−0.080	−0.143	−0.073	−0.024	−0.070	−0.013	−0.104	−0.279 14,15-EEQ −0.297 *
DiHETE tot	−0.065	−0.211 11,12-DiHETE −0.339 *	−0.003	−0.018	0.023	−0.089	−0.095	−0.086
EDP tot	−0.156	−0.090	−0.078	−0.084	−0.059	−0.072	−0.251 19,20-EDP −0.303 *	−0.259
DiHDPA tot	−0.018	−0.009	−0.094	−0.068	−0.050	−0.067	−0.280	−0.143

The table shows the coefficients of correlations (rs) between the metabolites of LA, AA, EPA, DHA via CYP450-epoxygenase/sEH and office and ambulatory BP in the whole sample and in the subgroups of HBP and NBP children. The underlined correlations remained significant after adjustment for sex, age, pubertal status and BMI. *: *p* < 0.05; ^: *p* < 0.01. NBP: normal blood pressure; HBP high blood pressure; SBP: systolic blood pressure; DBP: diastolic blood pressure; EpOME: epoxyoctadecenoic acid; DiHOME: dihydroxyoctadecenoic acid; EET: epoxyeicosatrienoic acid; DHET: dihydroxyeicosatrienoic acid; EEQ: epoxyeicosatetraenoic acid; DiHETE: dihydroxyeicosatetraenoic acid; EDP: epoxydocosapentaenoic acid; DiHDPA: dihydroxydocosapentaenoic acid.

**Table 5:** Correlations of LA, AA, EPA, DHA and the derived CYP450-epoxygenase/sEH metabolites with vascular tests in the whole sample and in the two individual subgroups of HBP and NBP obese children.

	cIMT	cIMT, (Percentile)	DC	DC, (Percentile)	FMD, %
**Whole population**					
LA	0.112	0.143	0.119	0.094	−0.150
AA	−0.142	−0.097	0.108	0.063	−0.005
EPA	−0.212	−0.305 *	−0.067	−0.015	0.176
DHA	−0.071	−0.170	−0.106	−0.053	0.139
EpOMEs	0.069	0.064	0.120	0.140	−0.141
DiHOMEs	−0.083	−0.077	0.095	0.085	−0.155
EETs	0.093	0.084	0.072	0.073	−0.185
DHETs	−0.070	−0.045	−0.058	−0.052	−0.049
EEQs	−0.184	−0.230 14,15-EEQ −0.279 * 17,18-EEQ −0.263 *	0.069	0.096	−0.008
DiHETEs	0.008	−0.026	0.012	0.024	0.176
EDPs	−0.082	−0.119	0.136	0.147	−0.158
DiHDPAs	−0.171	−0.206	0.091	0.121	−0.101
**HBP**					
LA	−0.121	−0.025	0.175	0.207	−0.400
AA	−0.172	−0.174	0.011	−0.039	−0.121
EPA	−0.304	−0.378	0.432	0.407	0.268
DHA	0.027	−0.002	−0.121	−0.136	−0.146
EpOMEs	−0.063	0.034	0.050	0.093	−0.050
DiHOMEs	0.067	0.071	−0.132	−0.132	0.004
EETs	−0.231	−0.061	0.314	0.214	0.118
DHETs	−0.301	−0.273	0.155	0.071	0.296
EEQs	−0.605 * 8;9-EEQ −0.493 * 14;15-EEQ −0.609 ^ 17;18-EEQ −0.615*	−0.620 ^ 8;9-EEQ −0.539 * 14;15-EEQ −0.657 ^ 17;18-EEQ −0.637 ^	0.374 11;12-EEQ 0.575 *	0.388 11;12-EEQ 0.582 *	0.288
DiHETEs	−0.354 5;6-DiHETE −0.559 *	−0.323 5;6-DiHETE −0.577 *	0.290	0.209	0.375 5;6-DiHETE −0.576 *
EDPs	−0.098	0.002	0.312	0.237	−0.220
DiHDPAs	−0.114	−0.104	0.148	0.086	−0.091
**NBP**					
LA	0.142	0.181	0.132	0.108	−0.082
AA	−0.067	−0.016	0.088	0.043	0.029
EPA	−0.243	−0.359 *	−0.174	−0.099	0.137
DHA	−0.097	−0.217	−0.137	−0.061	0.248
EpOMEs	0.095	0.078	0.107	0.140	−0.189
DiHOMEs	−0.163	−0.145	0.196	0.186	−0.209
EETs	0.159	0.101	0.032	0.068	−0.272
DHETs	0.010	0.025	−0.103	−0.091	−0.165
EEQs	−0.162	−0.235 14,15-EEQ −0.308 *	0.013	0.046	−0.116
DiHETEs	0.050	−0.014	−0.024	0.019	0.155
EDPs	−0.185	−0.247 19,20-EDP −0.296 *	0.081	0.121	−0.067
DiHDPAs	0.223	0.259	0.195	0.180	−0.095

The table shows the spearman coefficients of correlations (r_S_) between LA, AA, EPA, DHA and their metabolites via CYP450-epoxygenase/sEH with the vascular tests and their percentiles for sex and age in the whole sample and in the subgroups of HBP and NBP children. The underlined correlations remained significant after adjustment for sex, age, pubertal status and BMI. * *p*< 0.05; ^ *p* < 0.01. NBP: normal blood pressure; HBP high blood pressure; AA: arachidonic acid; DHA: docosahexaenoic acid; EPA: eicosapentaenoic acid; LA: linolenic acid; EpOME: epoxyoctadecenoic acid; DiHOME: dihydroxyoctadecenoic acid; EET: epoxyeicosatrienoic acid; DHET: dihydroxyeicosatrienoic acid; EEQ: epoxyeicosatetraenoic acid; DiHETE: dihydroxyeicosatetraenoic acid; EDP: epoxydocosapentaenoic acid; DiHDPA: dihydroxydocosapentaenoic acid; cIMT: carotid intima-media thickness; cDC: carotid distensibility; FMD: flow-mediated dilation.

## Supplementary Materials

The following are available online at http://www.mdpi.com/2072-6643/10/11/1689/s1, Table S1: General characteristics of the obese children divided according to pubertal status, Table S2: Correlations of CYP450-epoxygenase/sEH metabolites of LA, AA, EPA and DHA with BP percentiles in the whole sample and in the two subgroups of HBP and NBP obese children, Figure S1: Correlations between fatty acids and the estimated CYP450-epoxygenase activity.

## References

[1] Daniels S.R. (2009). Complications of obesity in children and adolescents. Int. J. Obes..

[2] Bonafini S., Fava C. (2017). Omega-3 fatty acids and cytochrome P450-derived eicosanoids in cardiovascular diseases: Which actions and interactions modulate hemodynamics?. Prostaglandins Other Lipid Mediat..

[3] Maffeis C., Pinelli L., Schutz Y. (1996). Fat intake and adiposity in 8 to 11 year-old obese children. Int. J. Obes..

[4] (1999). Dietary supplementation with n-3 polyunsaturated fatty acids and vitamin E after myocardial infarction: Results of the GISSI-Prevenzione trial. Gruppo Italiano per lo Studio della Sopravvivenza nell’Infarto miocardico. Lancet.

[5] Miller P.E., Van Elswyk M., Alexander D.D. (2014). Long-chain omega-3 fatty acids eicosapentaenoic acid and docosahexaenoic acid and blood pressure: A meta-analysis of randomized controlled trials. Am. J. Hypertens..

[6] Wang D.D., Li Y., Chiuve S.E., Stampfer M.J., Manson J.E., Rimm E.B., Willett W.C., Hu F.B. (2016). Association of Specific Dietary Fats with Total and Cause-Specific Mortality. JAMA Intern. Med..

[7] Vanhala M., Saltevo J., Soininen P., Kautiainen H., Kangas A.J., Ala-Korpela M., Mäntyselkä P. (2012). Serum omega-6 polyunsaturated fatty acids and the metabolic syndrome: A longitudinal population-based cohort study. Am. J. Epidemiol..

[8] Minihane A.M., Armah C.K., Miles E.A., Madden J.M., Clark A.B., Caslake M.J., Packard C.J., Kofler B.M., Lietz G., Curtis P.J. (2016). Consumption of Fish Oil Providing Amounts of Eicosapentaenoic Acid and Docosahexaenoic Acid That Can Be Obtained from the Diet Reduces Blood Pressure in Adults with Systolic Hypertension: A Retrospective Analysis. J. Nutr..

[9] Yang B., Shi M.-Q., Li Z.-H., Yang J.-J., Li D. (2016). Fish, Long-Chain n-3 PUFA and Incidence of Elevated Blood Pressure: A Meta-Analysis of Prospective Cohort Studies. Nutrients.

[10] Wang Q., Liang X., Wang L., Lu X., Huang J., Cao J., Li H., Gu D. (2012). Effect of omega-3 fatty acids supplementation on endothelial function: A meta-analysis of randomized controlled trials. Atherosclerosis.

[11] Tousoulis D., Plastiras A., Siasos G., Oikonomou E., Verveniotis A., Kokkou E., Maniatis K., Gouliopoulos N., Miliou A., Paraskevopoulos T. (2014). Omega-3 PUFAs improved endothelial function and arterial stiffness with a parallel antiinflammatory effect in adults with metabolic syndrome. Atherosclerosis.

[12] Pase M.P., Grima N.A., Sarris J. (2011). Do long-chain n-3 fatty acids reduce arterial stiffness? A meta-analysis of randomised controlled trials. Br. J. Nutr..

[13] Bonafini S., Tagetti A., Gaudino R., Cavarzere P., Montagnana M., Danese E., Benati M., Ramaroli D.A.D.A., Raimondi S., Giontella A. (2018). Individual fatty acids in erythrocyte membranes are associated with several features of the metabolic syndrome in obese children. Eur. J. Nutr..

[14] Schunck W.-H., Konkel A., Fischer R., Weylandt K.-H. (2018). Therapeutic potential of omega-3 fatty acid-derived epoxyeicosanoids in cardiovascular and inflammatory diseases. Pharmacol. Ther..

[15] Imig J.D. (2005). Epoxide hydrolase and epoxygenase metabolites as therapeutic targets for renal diseases. Am. J. Physiol. Ren. Physiol..

[16] Hercule H.C., Schunck W.-H.H., Gross V., Seringer J., Leung F.P., Weldon S.M., da Costa Goncalves A.C., Huang Y., Luft F.C., Gollasch M. (2009). Interaction between P450 eicosanoids and nitric oxide in the control of arterial tone in mice. Arterioscler. Thromb. Vasc. Biol..

[17] Cheng J., Ou J.-S., Singh H., Falck J.R., Narsimhaswamy D., Pritchard K.A., Schwartzman M.L. (2008). 20-Hydroxyeicosatetraenoic acid causes endothelial dysfunction via eNOS uncoupling. AJP Heart Circ. Physiol..

[18] Ishizuka T., Cheng J., Singh H., Vitto M.D., Manthati V.L., Falck J.R., Laniado-Schwartzman M. (2008). 20-Hydroxyeicosatetraenoic acid stimulates nuclear factor-kappaB activation and the production of inflammatory cytokines in human endothelial cells. J. Pharmacol. Exp. Ther..

[19] Mitchell L.A., Grant D.F., Melchert R.B., Petty N.M., Kennedy R.H. (2002). Linoleic acid metabolites act to increase contractility in isolated rat heart. Cardiovasc. Toxicol..

[20] Moran J.H., Nowak G., Grant D.F. (2001). Analysis of the toxic effects of linoleic acid, 12,13-cis-epoxyoctadecenoic acid, and 12,13-dihydroxyoctadecenoic acid in rabbit renal cortical mitochondria. Toxicol. Appl. Pharmacol..

[21] López-Vicario C., Alcaraz-Quiles J., García-Alonso V., Rius B., Hwang S.H., Titos E., Lopategi A., Hammock B.D., Arroyo V., Clària J. (2015). Inhibition of soluble epoxide hydrolase modulates inflammation and autophagy in obese adipose tissue and liver: Role for omega-3 epoxides. Proc. Natl. Acad. Sci. USA.

[22] López-vicario C., González-Périz A., Rius B., Morán-Salvador E., García-alonso V., Lozano J.J., Bataller R., Kang J.X., Arroyo V., Clària J. (2014). Molecular interplay between Δ5/Δ6 desaturases and long-chain fatty acids in the pathogenesis of non-alcoholic steatohepatitis. Gut.

[23] Agbor L.N., Walsh M.T., Boberg J.R., Walker M.K. (2012). Elevated blood pressure in cytochrome P4501A1 knockout mice is associated with reduced vasodilation to omega-3 polyunsaturated fatty acids. Toxicol. Appl. Pharmacol..

[24] Ye D., Zhang D., Oltman C., Dellsperger K., Lee H.-C.C., VanRollins M. (2002). Cytochrome p-450 epoxygenase metabolites of docosahexaenoate potently dilate coronary arterioles by activating large-conductance calcium-activated potassium channels. J. Pharmacol. Exp. Ther..

[25] Ulu A., Stephen Lee K.S., Miyabe C., Yang J., Hammock B.G., Dong H., Hammock B.D. (2014). An omega-3 epoxide of docosahexaenoic acid lowers blood pressure in angiotensin-II-dependent hypertension. J. Cardiovasc..

[26] Flynn J.T., Kaelber D.C., Baker-Smith C.M., Blowey D., Carroll A.E., Daniels S.R., de Ferranti S.D., Dionne J.M., Falkner B., Flinn S.K. (2017). Clinical Practice Guideline for Screening and Management of High Blood Pressure in Children and Adolescents. Pediatrics.

[27] Lurbe E., Agabiti-Rosei E., Cruickshank J.K., Dominiczak A., Erdine S., Hirth A., Invitti C., Litwin M., Mancia G., Pall D. (2016). 2016 European Society of Hypertension guidelines for the management of high blood pressure in children and adolescents. J. Hypertens..

[28] Wühl E., Witte K., Soergel M., Mehls O., Schaefer F. (2002). Distribution of 24-h ambulatory blood pressure in children: Normalized reference values and role of body dimensions. J. Hypertens..

[29] Sharma A.K., Metzger D.L., Daymont C., Hadjiyannakis S., Rodd C.J. (2015). LMS tables for waist-circumference and waist-height ratio Z-scores in children aged 5-19 y in NHANES III: Association with cardio-metabolic risks. Pediatr. Res..

[30] Barlow S.E. (2007). Expert Committee Expert committee recommendations regarding the prevention, assessment, and treatment of child and adolescent overweight and obesity: Summary report. Pediatrics.

[31] Cole T.J., Bellizzi M.C., Flegal K.M., Dietz W.H. (2000). Establishing a standard definition for child overweight and obesity worldwide: International survey. BMJ.

[32] Doyon A., Kracht D., Bayazit A.K., Deveci M., Duzova A., Krmar R.T., Litwin M., Niemirska A., Oguz B., Schmidt B.M.W. (2013). 4C Study Consortium Carotid artery intima-media thickness and distensibility in children and adolescents: Reference values and role of body dimensions. Hypertension.

[33] Urbina E.M., Williams R.V., Alpert B.S., Collins R.T., Daniels S.R., Hayman L., Jacobson M., Mahoney L., Mietus-Snyder M., Rocchini A. (2009). American Heart Association Atherosclerosis, Hypertension, and Obesity in Youth Committee of the Council on Cardiovascular Disease in the Young Noninvasive assessment of subclinical atherosclerosis in children and adolescents: Recommendations for standard assessment for clinical research: A scientific statement from the American Heart Association. Hypertension.

[34] Giannarelli C., Bianchini E., Bruno R.M., Magagna A., Landini L., Faita F., Gemignani V., Penno G., Taddei S., Ghiadoni L. (2012). Local carotid stiffness and intima-media thickness assessment by a novel ultrasound-based system in essential hypertension. Atherosclerosis.

[35] Arnold C., Markovic M., Blossey K., Wallukat G., Fischer R., Dechend R., Konkel A., von Schacky C., Luft F.C., Muller D.N. (2010). Arachidonic acid-metabolizing cytochrome P450 enzymes are targets of {omega}-3 fatty acids. J. Biol. Chem..

[36] Rivera J., Ward N., Hodgson J., Puddey I.B., Falck J.R., Croft K.D. (2004). Measurement of 20-hydroxyeicosatetraenoic acid in human urine by gas chromatography-mass spectrometry. Clin. Chem..

[37] Von Schacky C. (2011). The Omega-3 Index as a risk factor for cardiovascular diseases. Prostaglandins Other Lipid Mediat..

[38] Harris W.S., Von Schacky C. (2004). The Omega-3 Index: A new risk factor for death from coronary heart disease?. Prev. Med..

[39] Geleijnse J.M., Giltay E.J., Grobbee D.E., Donders A.R.T., Kok F.J. (2002). Blood pressure response to fish oil supplementation: Metaregression analysis of randomized trials. J. Hypertens..

[40] Bonafini S., Antoniazzi F., Maffeis C., Minuz P., Fava C. (2015). Beneficial effects of ω-3 PUFA in children on cardiovascular risk factors during childhood and adolescence. Prostaglandins Other Lipid Mediat..

[41] Damsgaard C.T., Schack-Nielsen L., Michaelsen K.F., Fruekilde M.B., Hels O., Lauritzen L. (2006). Fish oil affects blood pressure and the plasma lipid profile in healthy Danish infants. J. Nutr..

[42] Damsgaard C.T., Eidner M.B., Stark K.D., Hjorth M.F., Sjödin A., Andersen M.R., Andersen R., Tetens I., Astrup A., Michaelsen K.F., Lauritzen L. (2014). Eicosapentaenoic acid and docosahexaenoic acid in whole blood are differentially and sex-specifically associated with cardiometabolic risk markers in 8-11-year-old danish children. PLoS ONE.

[43] Capdevila J., Wang W. (2013). Role of cytochrome P450 epoxygenase in regulating renal membrane transport and hypertension. Curr. Opin. Nephrol. Hypertens..

[44] Minuz P., Jiang H., Fava C., Turolo L., Tacconelli S., Ricci M., Patrignani P., Morganti A., Lechi A., McGiff J.C. (2008). Altered release of cytochrome p450 metabolites of arachidonic acid in renovascular disease. Hypertension.

[45] Dalle Vedove F., Fava C., Jiang H., Zanconato G., Quilley J., Brunelli M., Guglielmi V., Vattemi G., Minuz P. (2016). Increased epoxyeicosatrienoic acids and reduced soluble epoxide hydrolase expression in the preeclamptic placenta. J. Hypertens..

[46] Jiang H., McGiff J.C., Fava C., Amen G., Nesta E., Zanconato G., Quilley J., Minuz P. (2013). Maternal and fetal epoxyeicosatrienoic acids in normotensive and preeclamptic pregnancies. Am. J. Hypertens..

[47] Taddei S., Versari D., Cipriano A., Ghiadoni L., Galetta F., Franzoni F., Magagna A., Virdis A., Salvetti A. (2006). Identification of a cytochrome P450 2C9-derived endothelium-derived hyperpolarizing factor in essential hypertensive patients. J. Am. Coll. Cardiol..

[48] Halcox J.P., Narayanan S., Cramer-Joyce L., Mincemoyer R., Quyyumi A.A. (2001). Characterization of endothelium-derived hyperpolarizing factor in the human forearm microcirculation. Am. J. Physiol. Heart Circ. Physiol..

[49] Lee C.R., Imig J.D., Edin M.L., Foley J., DeGraff L.M., Bradbury J.A., Graves J.P., Lih F.B., Clark J., Myers P. (2010). Endothelial expression of human cytochrome P450 epoxygenases lowers blood pressure and attenuates hypertension-induced renal injury in mice. FASEB J..

[50] Virdis A., Cetani F., Giannarelli C., Banti C., Ghiadoni L., Ambrogini E., Carrara D., Pinchera A., Taddei S., Bernini G. (2010). The Sulfaphenazole-Sensitive Pathway Acts as a Compensatory Mechanism for Impaired Nitric Oxide Availability in Patients with Primary Hyperparathyroidism. Effect of Surgical Treatment. J. Clin. Endocrinol. Metab..

[51] Morris M.C., Sacks F., Rosner B. (1993). Does fish oil lower blood pressure? A meta-analysis of controlled trials. Circulation.

[52] Lynes M.D., Leiria L.O., Lundh M., Bartelt A., Shamsi F., Huang T.L., Takahashi H., Hirshman M.F., Schlein C., Lee A. (2017). The cold-induced lipokine 12,13-diHOME promotes fatty acid transport into brown adipose tissue. Nat. Med..

[53] Goodfriend T.L., Ball D.L., Egan B.M., Campbell W.B., Nithipatikom K. (2004). Epoxy-Keto Derivative of Linoleic Acid Stimulates Aldosterone Secretion. Hypertension.

[54] Caligiuri S.P.B., Love K., Winter T., Gauthier J., Taylor C.G., Blydt-Hansen T., Zahradka P., Aukema H.M. (2013). Dietary linoleic acid and α-linolenic acid differentially affect renal oxylipins and phospholipid fatty acids in diet-induced obese rats. J. Nutr..

[55] Hamazaki K., Iso H., Eshak E.S., Ikehara S., Ikeda A., Iwasaki M., Hamazaki T., Tsugane S., Grp J.S. (2018). Plasma levels of n-3 fatty acids and risk of coronary heart disease among Japanese: The Japan Public Health Center-based (JPHC) study. Atherosclerosis.

[56] Harris W.S., Del Gobbo L., Tintle N.L. (2017). The Omega-3 Index and relative risk for coronary heart disease mortality: Estimation from 10 cohort studies. Atherosclerosis.

