# Supplementary material for: Possible Role of CYP450 Generated Omega-3/Omega-6 PUFA Metabolites in the Modulation of Blood Pressure and Vascular Function in Obese Children
Source: Nutrients. 2018 Nov 5;10(11):1689. doi: 10.3390/nu10111689 (PMC6267577; doi:10.3390/nu10111689)
Supplement: Supplementary file 1 [file nutrients-10-01689-s001.zip › nutrients-378401-supplementary-latest/Figure S1-09-10-18.docx]

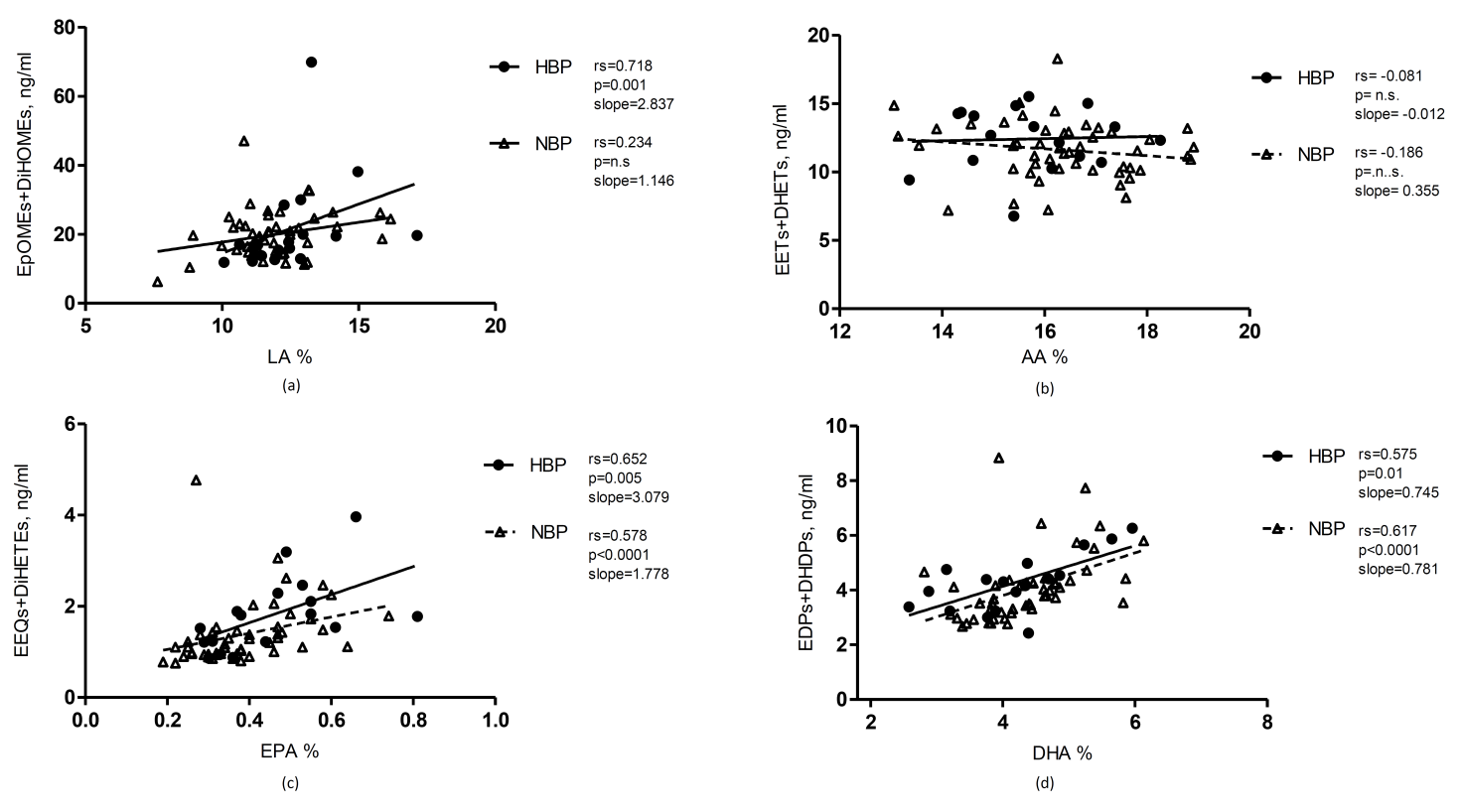


**Figure S1.** Correlations between fatty acids and the estimated CYP450-epoxygenase activity. The two subgroups of obese children with high (HBP) and normal blood pressure (NBP) are differently graphically depicted. The panels represet: **(a)** the correlation of LA with the estimated activity of CYP450 epoxygenase in metabolizing LA, as calculated as the sum of EpOMEs plus DiHOMEs; **(b)** the association of AA the estimated activity of CYP450 epoxygenase in metabolizing AA, as calcuclated as the sum of EETs plus DHETs; **(c)** the correlation of EPA the estimated activity of CYP450 epoxygenase in metabolizing EA, as caluclated as the sum of EEQs plus DiHETEs**; (d)** the association of DHA the estimated activity of CYP450 epoxygenase in metabolizing DHA, as calculated as the sum of EDPs plus DHDPs.

LA: Linoleic acid; AA: Arachidonic acid; EPA: Eicosapentaenoic acid; DHA: Docosahexaenoic acid; ; EpOME: epoxyoctadecenoic acid; DiHOME: dihydroxyoctadecenoic acid; EET: epoxyeicosatrienoic acid; DHET: dihydroxyeicosatrienoic acid; EEQ: epoxyeicosatetraenoic acid; DiHETE: dihydroxyeicosatetraenoic acid; EDP: epoxydocosapentaenoic acid; DiHDPA: dihydroxydocosapentaenoic acid.; HBP: high blood pressure subgroup; NBP: normal blood pressure subgroup.
