# Supplementary material for: Possible Role of CYP450 Generated Omega-3/Omega-6 PUFA Metabolites in the Modulation of Blood Pressure and Vascular Function in Obese Children
Source: Nutrients. 2018 Nov 5;10(11):1689. doi: 10.3390/nu10111689 (PMC6267577; doi:10.3390/nu10111689)
Supplement: Supplementary file 1 [file nutrients-10-01689-s001.zip › nutrients-378401-supplementary-latest/Table S1-24-10-2018.docx]

**Table S1.** General characteristics of the obese children divided according to pubertal status.

|  | **Pre-Pubertal**  **(n=36)** | **Pubertal**  **(n=30)** |  |
| --- | --- | --- | --- |
| **Variable** | **Median (rangeIQ)** | **Median (rangeIQ)** | **p-value*** |
| **Age, y** | 10.0 (9.0 - 11.0) | 13.0 (12.0 - 15.0) | **<0.001°** |
| **BMI, kg/m^2^**  **BMI percentile** | 28.1 (25.2 - 30.3)  98.7 (97.8 - 99.3) | 30.2 (26.4 - 33.9)  98.0 (96.9 - 99.0) | **0.033**  0.064 |
| **Office-SBP, mmHg**  **Office-SBP percentile** | 114.5 (109.8 - 121.9)  79.4 (64.2 - 96.5) | 122.7 (115.2 - 129.8)  86.1 (71.4 - 95.8) | **0.016**  0.616 |
| **Office-DBP, mmHg**  **Office-DBP percentile** | 66.5 (62.6 - 72.8)  65.6 (54.0 - 80.0) | 68.2 (64.3 - 76.4)  65.2 (42.9 - 80.2) | 0.252  0.511 |
| **24 hour-SBP, mmHg**  **24 hour-SBP, percentile** | 115.0 (109.3 - 121.0)  75.6 (51.7 - 86.8) | 117.5 (111.5 - 120.00)  51.5 (33.8 - 74.6) | 0.466  **0.015** |
| **24 hour-DBP, mmHg**  **24 hour-DBP percentile** | 67.0 (63.3 - 70.0)  51.8 (28.9 - 73.5) | 65.0 (62.0 - 69.0)  35.1 (16.8 - 60.9) | 0.225  0.127 |
| **cIMT,mm**  **cIMT percentile** | 0.46 (0.41 - 0.51)  98.4 (80.4 - 80.4) | 0.43 (0.40 - 0.48)  86.3 (58.8 - 97.2) | 0.241  **0.033** |
| **cDC, 10^-3^/Kpa**  **cDC percentile** | 43.3 (35.9 - 49.7)  11.9 (4.0 - 27.9) | 39.0 (32.6 - 44.2)  10.5 (2.1 - 18.8) | 0.096  0.290 |
| **FMD, %** | 7.0 (3.8 - 9.7) | 7.9 (3.8 - 10.5) | 0.466 |
| **Glucose,mg/dL** | 88 (83.5 - 90) | 86 (82- 94.25) | 0.643 |
| **Insulin,uU/** | 17.5 (11.8 – 24.2) | 21.8 (15.4 – 28.7) | 0.104 |
| **Cholesterol, mg/dL** | 163.5 (137.5 - 185) | 157 (138 - 193) | 0.956 |
| **Triglycerides, mg/dL** | 85 (66 - 109) | 69 (52 – 98.5) | 0.195 |
| **LA, %** | 12.2 (11.1 - 13.1) | 11.4 (10.9 - 12.4) | 0.080 |
| **AA, %** | 16.2 (15.4 - 17.5) | 16.2 (15.5 - 16.8) | 0.680 |
| **EPA, %** | 0.37 (0.28 - 0.47) | 0.39 (0.33 - 0.56) | 0.061 |
| **DHA, %** | 3.9 (3.5 - 4.4) | 4.8 (4.2 - 5.3) | **<0.001°** |
| **Omega-3 Index, %** | 4.3 (3.8 - 4.47) | 5.1 (4.5 - 5.7) | **<0.001°** |
| **EpOMEs, ng/mL** | 11.2 (8.7 - 15.7) | 10.0 (8.0 - 15.9) | 0.479 |
| **DiHOMEs, ng/mL** | 7.6 (5.9 - 10.0) | 6.0 (4.8 - 8.1) | **0.020** |
| **EpOMEs/DiHOMEs** | 1.4 (0.9 - 2.1) | 1.7 (1.3 - 2.3) | 0.140 |
| **EpOMEs+DiHOMEs** | 19.9 (15.4 - 26.0) | 17.3 (14.5 - 22.0) | 0.110 |
| **EETs, ng/mL** | 7.6 (6.7 - 8.9) | 7.6 (6.8 - 8.9) | 0.857 |
| **DHETs, ng/mL** | 4.0 (3.4 - 4.5) | 4.0 (3.4 - 4.9) | 0.515 |
| **EETs/DHETs** | 19 (1.6 - 2.3) | 1.9 (1.5 - 2.2) | 0.643 |
| **EETs+DHETs** | 11.9 (10.3 - 13.3) | 11.9 (10.5 - 13.3) | 0.728 |
| **EEQs, ng/mL** | 0.4 (0.2 - 0.5) | 0.4 (0.3 - 0.5) | 0.584 |
| **DiHETEs, ng/mL** | 0.9 (0.7 - 1.3) | 0.9 (0.7 - 1.2) | 0.661 |
| **EEQs/DiHETEs** | 0.34 (0.30 - 0.43) | 0.4 (0.3 - 0.5) | 0.096 |
| **EEQs+DiHETEs** | 1.3 (1.0 - 1.8) | 1.3 (1.0 - 1.7) | 0.974 |
| **EDPs, ng/mL** | 2.9 (2.3 - 3.4) | 3.1 (2.6 - 4.4) | 0.068 |
| **DiHDPAs, ng/mL** | 0.8 (0.7 - 1.0) | 1.0 (0.8 - 1.2) | **0.001°** |
| **EDPs/DiHDPAs** | 3.4 (3.0 - 3.8) | 3.3 (2.8 - 4.0) | 0.676 |
| **EDPs+DiHDPAs** | 3.7 (3.0 - 4.3) | 4.2 (3.5 - 5.7) | **0.020** |
| **20-HETE,ng/mL** | 0.71 (0.61 – 0.88) | 0.63 (0.57 – 0.83) | 0.495 |
| **22-HDHA, ng/mL** | 0.68 (0.41 – 0.87) | 0.71 (0.48 – 0.91) | 0.268 |

Children were classified as pubertal or pre-pubertal according with Tanner stage.

BMI: body mass index - SBP: systolic blood pressure - DBP: diastolic blood pressure - cIMT: carotid intima-media thickness - cDC: carotid distensibility coefficient - FMD: flow-mediated dilation - LA: Linoleic acid - AA: Arachidonic acid - EPA: Eicosapentaenoic acid - DHA: Docosahexaenoic acid - Omega-3 Index: ( EPA + DHA)/total FA*100 - EpOME: epoxyoctadecenoic acid - DiHOME: dihydroxyoctadecenoic acid - EET: epoxyeicosatrienoic acid - DHET: dihydroxyeicosatrienoic acid - EEQ: epoxyeicosatetraenoic acid - DiHETE: dihydroxyeicosatetraenoic acid - EDP: epoxydocosapentaenoic acid - DiHDPA: dihydroxydocosapentaenoic acid - 20-HETE: 20-hydroxyeicosatetraenoic acid – 22-HDHA: 22-hydroxydocosahexaenoic acid . *Mann-Whitney U Test,° p< 0.05 after False Discovery Rate correction
