# Supplementary material for: Possible Role of CYP450 Generated Omega-3/Omega-6 PUFA Metabolites in the Modulation of Blood Pressure and Vascular Function in Obese Children
Source: Nutrients. 2018 Nov 5;10(11):1689. doi: 10.3390/nu10111689 (PMC6267577; doi:10.3390/nu10111689)
Supplement: Supplementary file 1 [file nutrients-10-01689-s001.zip › nutrients-378401-supplementary-latest/Table S2.docx]

**Table S2.** Correlations of CYP450-epoxygenase/sEH metabolites of LA, AA, EPA and DHA with BP percentiles in the whole sample and in the two subgroups of HBP and NBP obese children.

|  | **Office SBP,**  **percentile** | **Office DBP,**  **percentile** | **24-h SBP,**  **percentile** | **24-h DBP,**  **percentile** | **Day-time SBP,**  **percentile** | **Day-time DBP,**  **percentile** | **Night-timeSBP,**  **percentile** | **Night-timeDBP,**  **percentile** |
| --- | --- | --- | --- | --- | --- | --- | --- | --- |
| **Whole population** | | | | | | | | |
| **EpOMEtot** | -0.145 | 0.045 | -0.028 | 0.058 | -0.016 | 0.068 | -0.086 | 0.001 |
| **DiHOME tot** | 0.011 | **0.344^**  **9,10-DiHOME 0.370^** | 0.095 | 0.149 | 0.002 | 0.048 | 0.156 | **0.278***  **9,10-DiHOME 0.280*** |
| **EET tot** | 0.036 | -0.029 | -0.004 | 0.028 | -0.012 | 0.032 | -0.041 | -0.038 |
| **DHET tot** | 0.126 | 0.035 | 0.028 | 0.012 | 0.063 | 0.060 | -0.040 | -0.049 |
| **EEQ tot** | -0.186 | -0.180  **8,9-EEQ -0.258*** | 0.061 | 0.026 | 0.059 | 0.022 | 0.041 | -0.067 |
| **DiHETE tot** | -0.114  **17,18-DiHETE -0.492*** | -0.116 | 0.111 | 0.097 | 0.155 | 0.059 | 0.055 | 0.089 |
| **EDP tot** | -0.219 | -0.190 | -0.055 | -0.075 | -0.011 | -0.060 | -0.109 | -0.110 |
| **DiHDPA tot** | -0.177 | -0.133 | -0.153 | -0.083 | -0.052 | -0.034 | -0.211 | -0.146 |
| **HBP** | | | | | | | | |
| **EpOME tot** | -0.297 | -0.054 | -0.088 | -0.299 | -0.181 | -0.404 | -0.075 | 0.113 |
| **DiHOME tot** | -0.059 | 0.157 | -0.252 | -0.096 | -0.250 | -0.123 | -0.251 | 0.054 |
| **EET tot** | -0.069 | -0.025 | 0.020 | 0.115 | 0.069 | 0.191 | -0.063 | -0.002 |
| **DHET tot** | 0.009 | -0.296 | -0.044 | -0.056 | -0.011 | 0.054 | -0.082 | -0.124 |
| **EEQ tot** | **-0.536***  **11,12-EEQ -0.670^**  **17,18-EEQ -0.615^** | -0.352 | -0.135 | -0.129 | -0.027 | -0.069 | -0.152  **17,18-EEQ -0.615^** | -0.223 |
| **DiHETE tot** | -0.346 | -0.196 | -0.139 | 0.131 | -0.026 | 0.188 | -0.137 | 0.021 |
| **EDP tot** | -0.273 | -0.263 | 0.189 | -0.231 | 0.248 | -0.192 | -0.073 | -0.278 |
| **DiHDPA tot** | -0.120 | -0.428 | 0.139 | -0.167 | 0.221 | -0.045 | -0.122 | -0.416 |
| **NBP** | | | | | | | | |
| **EpOME tot** | -0.094 | 0.142 | 0.003 | 0.140 | 0.061 | 0.200 | -0.123 | -0.004 |
| **DiHOME tot** | -0.010 | **0.375^**  **9,10-DiHOME 0.394^** | 0.091 | 0.153 | -0.033 | 0.052 | 0.203 | **0.352***  **9,10-DiHOME 0.349***  **12,13-DiHOME 0.294*** |
| **EET tot** | -0.040 | -0.091 | -0.171 | -0.108 | -0.145 | -0.080 | -0.280 | -0.244  **8,9-EET -0.294*** |
| **DHET tot** | 0.169 | 0.053 | -0.059 | 0.013 | -0.025 | 0.039 | -0.151 | -0.099 |
| **EEQ tot** | -0.154 | -0.138 | -0.118 | -0.038 | -0.093 | -0.010 | -0.167 | -0.290  **11,12-EEQ -0.299** |
| **DiHETE tot** | -0.155  **11,12-DiHETE -0.348*** | -0.178  **11,12-DiHETE -0.284*** | -0.105 | -0.050 | -0.028 | -0.093 | -0.186 | -0.124 |
| **EDP tot** | **-0.347***  **7,8-EDP -0.307***  **10,11-EDP -0.327***  **13,14-EDP -0.379^**  **16,17-EDP -0.339***  **19,20-EDP -0.360*** | -0.181 | -0.281  **7,8-EDP -0.323***  **19,20-EDP -0.314*** | -0.105 | -0.185 | -0.063 | **-0.440^**  **7,8-EDP -0.447^**  **10,11-EDP -0.418^**  **13,14-EDP -0.420^**  **16,17-EDP -0.376***  **19,20-EDP -0.460^** | -0.224 |
| **DiHDPA tot** | -0.232 | -0.139 | **-0.313*** | -0.079 | -0.174 | -0.042 | **-0.412^** | -0.187 |

The table shows the coefficients of correlations (r_S_) between the metabolites of LA, AA, EPA, and DHA via CYP450-epoxygenase/sEH and office and ambulatory BP percentile for sex, age and height in the whole sample and in the subgroups of HBP and NBP children. The underlined correlations remained significant after adjustment for sex, age, pubertal status and BMI. * p< 0.05; ^ p< 0.01; ° p< 0.05 after False Discovery Rate correction.

NBP: normal blood pressure; HBP high blood pressure; SBP: systolic blood pressure; DBP: diastolic blood pressure; EpOME: epoxyoctadecenoic acid; DiHOME: dihydroxyoctadecenoic acid; EET: epoxyeicosatrienoic acid; DHET: dihydroxyeicosatrienoic acid; EEQ: epoxyeicosatetraenoic acid; DiHETE: dihydroxyeicosatetraenoic acid;

EDP: epoxydocosapentaenoic acid; DiHDPA: dihydroxydocosapentaenoic acid.
